# Family-Environmental Factors Associated with Attention Deficit Hyperactivity Disorder in Chinese Children: A Case-Control Study

**DOI:** 10.1371/journal.pone.0050543

**Published:** 2012-11-28

**Authors:** Xianming du Prel Carroll, Honggang Yi, Yuezhu Liang, Ke Pang, Sandra Leeper-Woodford, Patrizia Riccardi, Xianhong Liang

**Affiliations:** 1 Department of Social Medicine and Health Education, School of Public Health, Nanjing Medical University, Nanjing, China; 2 Department of Epidemiology and Biostatistics, School of Public Health, Nanjing Medical University, Nanjing, China; 3 Beijing Children and Adolescents Mental Health Center, Beijing An Ding Hospital, Capital Medical University, Beijing, China; 4 Department of Neurology, Beijing Tian Tan Hospital, Capital Medical University, Beijing, China; 5 Division of Basic Medical Sciences, Mercer University School of Medicine, Macon, Georgia, United States of America; 6 Department of Psychiatry, Mercer University School of Medicine, Macon, Georgia, United States of America; Pennsylvania State University, United States of America

## Abstract

**Background:**

Attention deficit hyperactivity disorder (ADHD) is one of the most common psychiatric disorders, affecting an estimated 5 to 12% of school-aged children worldwide. From 15 to 19 million Chinese children suffer from ADHD. The aim of this study was to investigate the association between family-environmental factors and ADHD in a sample of Chinese children.

**Methods:**

A pair-matched, case-control study was conducted with 161 ADHD children and 161 non-ADHD children of matching age and sex, all from 5–18 years of age. The ADHD subjects and the normal controls were all evaluated via structured diagnostic interviews. We examined the association between family-environmental factors and ADHD using the conditional multiple logistic regression with backward stepwise selection to predict the associated factors of ADHD.

**Results:**

Having experienced emotional abuse and being a single child were both significant factors associated with children diagnosed with ADHD. ADHD subjects were more likely to have suffered from emotional abuse (OR = 11.09, 95% CI = 2.15–57.29, P = 0.004) and have been a single child in the family (OR = 6.32, 95% CI = 2.09–19.14, P = 0.001) when compared to normal controls. The results were not modified by other confounding factors.

**Conclusion:**

Our findings provide evidence that family-environmental factors are associated with ADHD among children in China. These findings, if confirmed by future research, may help to decrease ADHD by increasing the awareness of the effects of childhood emotional abuse.

## Introduction

Attention deficit hyperactivity disorder (ADHD) is one of the most common psychiatric disorders occurring during childhood and adolescence, affecting an estimated 5 to 12% of school-aged children worldwide [1]–[3]. Children with ADHD may suffer from cognitive and social deficits, in addition to displaying behavioral problems that result in disturbances in peer and family relationships, as well as poor academic achievement [4], [5]. Compared with the current data supporting the roles of genetic and biological factors in the etiology of ADHD, research on environmental, social and interpersonal aspects is less robust [6], [7].

Family structure, such as being a single child in a family with either two biological parents or with a single parent/step-parent, may play a role in child psychiatric disorders since these factors affect material resources and emotional strain in families [8], [9]. In a review of the family factors associated with ADHD, Johnston and Mash highlighted disturbances in family functioning, conflicted parent–child relationships, as well as increased parenting stress and psychopathology as common co-occurring factors [10], [11]. Previous investigations [12] have also found that child abuse and parental psychiatric disorders are associated with higher rates of ADHD with comorbid disruptive behavior disorder. While uncovering susceptibility genes for ADHD may help us understand the emergence of ADHD symptoms, researchers have speculated that unique environmental factors may play a larger role in determining outcomes for children, even if they are not a primary cause of the core symptoms [11], [13]. Understanding the risk and protective factors within the environment, such as the influence of the family, school and community, as well as their interactions with child characteristics, can extend the findings of genetic work in tracing the variability in development of children with ADHD.

A 3 year panel study in Taiwan [14] investigated adolescent mental disorder and showed that the most prevalent psychiatric condition among 7th and 8th graders was ADHD (weighted prevalence 7.5% and 6.1%). A recent survey [15] in China showed that a total of 15 to 19 million Chinese children suffer from ADHD, therefore indicating that ADHD has become a serious public health problem in China. As in many other countries today, youth in China are also facing greater familial-socio environmental stress than their predecessors. Owing to the rigid educational system and high parental expectation on academic achievement, the competition in joint entrance examinations for junior/senior high schools and universities is very keen [16]. These family-environmental changes and their impact on adolescent psychopathology deserve an adequate inquiry. However, studies on the relationship between ADHD and the family environment, with respect to how demographic variables interact with family factors, are lacking. The aim of this study is to investigate the relationship between family-environmental factors and ADHD in a sample of Chinese children. Our hypothesis is that these factors are related to the ADHD children and their family environment when compared to normal controls.

## Methods

### Study Subjects

This study was designed as a pair matching case-control study and conducted from July 2009 to May 2010. ADHD subjects were recruited from the Beijing Children and Adolescents Mental Health Center at Beijing An Ding Hospital of Capital Medical University in Beijing, China. All ADHD (ICD-10 codes F90, 208–210) subjects were children of Chinese Han nationality and between 5 to 18 years old at the time of the investigation. All ADHD participants were evaluated by child psychiatrists and fully met DSM-IV-TR criteria for ADHD (any subtype) [17]. The DSM-IV-TR diagnostic criteria were previously translated into Chinese, and the reliability for the ADHD diagnosis was previously assessed [15], [18]–[20]. The diagnoses of ADHD were derived from a structured diagnostic interview based on Schedule for Affective Disorders and Schizophrenia for School-Age Children (K-SADS-E) [21], which was modified to assess DSM-IV-TR criteria and incorporate parents’ and teachers’ reports of behavioral symptoms, clinical observation of behavior, the Aberrant Behavior Checklist [22], and tests of attention such as the Conners Continuous Performance Test [23]. One on one interviews of the ADHD participants, at least one of their parents/primary caregivers, and their teachers were conducted by trained researchers who are child/adolescent psychiatrists or psychologists in the outpatient department of the Beijing Children and Adolescents Mental Health Center. These diagnoses were then independently reassessed by two senior psychiatrists, who systematically reviewed all of the interview records. In the reassessment, the principle of rate-down was employed, and any information that was dubious or uncertain was discarded. Psychiatric diagnoses generated from this reassessment were jointly discussed, and a consensus diagnosis was taken as final. Exclusion criteria of the ADHD subjects included the presence of any other psychiatric illness such as depression, autism, Asperger syndrome, other pervasive developmental disorders (ICD-10 codes F84.0–F84.9, 308.0), and mental retardation (ICD-10 codes F70–F79, 312–315) [20].

Normal controls were children between 5 to 18 years old randomly selected from local elementary and middle/high schools during the same study period and from the same district in Beijing as the ADHD subjects. Using the pair-matched test design, each ADHD subject and normal control had the same sex and the same age (difference between birthdays within 6 months). The same exclusion criteria applied to the ADHD subjects was applied to the normal controls.

The study was approved by the institutional review boards of An Ding Hospital of Capital Medical University and Mercer University, and complied with all applicable requirements of the United States. Written informed consent was obtained from each of the parents after the purpose and procedure of the study was explained. Parents and teachers of the ADHD subjects completed the questionnaire with a participation rate of 97.5%, and parents and teachers of the normal controls completed the questionnaire with a participation rate of 100%.

### Measures

#### Matched factors

To address important confounding factors, such as the male sex and younger ages being associated with an increased prevalence of ADHD [24], [25], we designed pair-matches on age and sex for the control vs. ADHD subjects in this study. Therefore the control subjects were exposed to associated factors at the same sex and age as the ADHD subjects.

#### Biological factors

The variable of maternal stress during pregnancy was provided by the biological mothers of both the ADHD and normal subjects. The mothers were asked whether or not they had experienced any of 10 major life stress events selected from a broader list of life stress events [26], [27]: pregnancy problems, death of a close friend or relative, separation or divorce, marital problems, problems with children, job loss (involuntary), partner’s job loss (involuntary), monetary problems, residential relocation, or any other stressful events. If the answer they provided was one or more positive response to the 10 major events during their pregnancy, the coding would be a ‘yes’, otherwise ‘no’. Pregnancy-induced hypertension (PIH) information was also provided by the affected mothers of the study subjects. We analyzed continuous potential factors, such as maternal age at the child’s birth, according to biological rationales (median split) as categorical variables: ≤26 years, and >26 years of age. The data of our study sample showed that none of the mothers smoked or drank during pregnancy, so the variables of maternal smoking and maternal drinking were not considered for further analysis.

#### Family-environmental factors

In the questionnaire, the parents were required to provide information about familial factors present during the child’s lifetime. Maternal and paternal education was assigned as being either compulsory education (≤9 years), high school education (9–12 years), or some college or advanced training (≥12 years) [20]. The number of siblings was asked to determine whether or not the child was a single child [28]. Family structure was divided into 2 categories [29]: the child having an intact family with both biological parents, and the child having other family structures (which included single-parent and 2-parent families, in which one or both parents were step-parents). Since literature suggests that family conflicts are connected with children functioning [30], [31], the parents informed in a yes/no format in regards to the occurrence of conflicts between adult family members/relatives. Emotional abuse was defined by two questions from the Conflict Tactics Scale (CTS) [32]. The questions, which were asked to the ADHD subjects, were as follows: 1) “How often did a parent, stepparent, or adult living in your home swear at you, insult you, or put you down?” 2) “How often did a parent, stepparent, or adult living in your home act in a way that made you afraid that you might be physically hurt?” Responses of “often” or “very often” to either item defined emotional abuse during childhood.

#### Lifestyle factors

Altogether, seven lifestyle variables were included in this study. Parents were asked about their children’s exposure to domestic tobacco smoke and domestic alcohol consumption. Study factors were defined as binary variables - that is, domestic tobacco smoke (yes/no) and domestic alcohol consumption (yes/no) [33], [34]. Based on questions included on the 2009 US middle school Youth Risk Behavior Survey (YRBS) [35], parents and teachers were asked to report physical activity of the study subjects (“During the past 7 days, on how many days was the child physically active for a total of at least 60 minutes per day?”). The answers were divided into the following: physical inactive, outdoor activity ≤3 days in 1 week; physical active, outdoor activity >3 days in 1 week. Parents also reported on the number of hours that their child viewed television and accessed the internet. The American Academy of Pediatrics recommends that children ≥2 years of age limit their time with entertainment media to ≤1 to 2 hours of programming daily [36], [37]. The measure of interest was >2 hours of television viewing (including videos) and internet accessing daily. The behavioral factor for accidental injury was defined as a binary variable (yes/no), and a nutritional factor was also included in the questionnaire and defined as daily dietary supplement intake (yes/no).

### Statistical Analyses

Statistical analyses were performed by SAS, version 9.2 (SAS Institute Inc., Cary, NC, USA). To assess the impact of the associated factors on the dependent variable-ADHD, we performed multivariate logistic regression analysis with all factors simultaneously included in the same model to adjust each other. The following independent variables were included in the model: age, sex, biological factors, family-environmental factors, and lifestyle factors. Finally, we performed a conditional logistic regression analysis with backward stepwise procedures based on the maximum partial likelihood estimates to construct a final best fit logistic regression model to identify the predictors of risk for ADHD among all factors. This specifies the significance level at p 0.05 for entering an explanatory variable into the model in the backward stepwise method. We estimated odds ratios (ORs) and 95% confidence intervals (CIs) for differing levels of exposure. All statistical tests were considered to be significant at an alpha level of 0.05 on a two-tailed test.

## Results

The analysis included 161 ADHD cases and 161 non-ADHD control subjects matched by age and sex. There were 113 boys and 48 girls included in the case group and control group of this study. There was no statistical age difference between the two groups (mean age of 12.89±2.96 in the case group and mean age of 12.91±2.81 in the control group).

### Demographic and Distribution of Biological Factors, Family-environmental Factors, and Lifestyle Factors

General information in demographic and distribution of biological factors is shown in Table 1. We found that ADHD subjects were significantly associated with maternal stress during pregnancy (OR = 3.67, 95% CI = 1.49–9.04, p = 0.005).

**Table 1:** Demographic and Distribution of Biological Factors of ADHD: Comparisons of ADHD Cases and Non-ADHD Control subjects.

Characteristic	ADHD (n = 161)	Control (n = 161)	p-Value	OR (95% CI)
***Matched Factors***				
Age (years)	12.89±2.96	12.91±2.81	0.675	–
Sex				
Male	113(70.19%)	113(70.19%)		
Female	48(29.81%)	48(29.81%)	–	–
***Biological Factors***				
Maternal age at childbirth (years)				
≤26	98 (60.87%)	101(62.73%)		
>26	63(39.13%)	60(37.27%)	0.740	1.08(0.69–1.68)
Maternal stress during pregnancy				
No	136(86.08%)	152(96.20%)		
Yes	22(13.92%)	6(3.80%)	**0.005**	**3.67(1.49–9.04)**
Pregnancy induced hypertension				
No	147(94.84%)	146(94.81%)		
Yes	8(5.16%)	8(5.19%)	1.000	1.00(0.35–2.85)

–, no data.

P-value and OR (95% CI) were obtained from the multivariate logistic regression model that simultaneously included biological factors.

ORs for ADHD cases and controls by family-environmental factors are presented in Table 2. ADHD subjects were significantly associated with single-child (OR = 4.00, 95% CI = 2.27–7.044, p<.0001), family conflicts (OR = 3.09, 95% CI = 1.57–6.10, p = 0.001), presence of emotional abuse (OR = 10.50, 95% CI = 3.77–29.28, p<.0001), and paternal education (OR = 2.07, 95% CI = 1.07–4.01, p = 0.017) when the 9–12 years education group was compared with the ≥12 years education group.

**Table 2:** Distribution of Family-Environmental Factors of ADHD: Comparisons of ADHD Cases and Non-ADHD Control Subjects.

Characteristic	ADHD(n = 161)	Control(n = 161)	p-Value	OR (95% CI)
Maternal Education (Years)				
≥12	36(22.36%)	42(26.58%)		
9–12	50(31.06%)	39(24.68%)	0.184	1.50(0.81–2.76)
≤9	75(46.58%)	77(47.83%)	0.744	1.14(0.66–1.96)
Paternal Education (Years)				
≥12	33(20.63%)	41(25.79%)		
9–12	45(28.13%)	27(16.98%)	**0.017**	**2.07(1.07–4.01)**
≤9	82(51.25%)	91(57.23%)	0.270	1.12(0.65–1.93)
Single Child				
No	22(13.66%)	66(41.77%)		
Yes	139(86.34%)	92(58.23%)	**<.0001**	**4.00(2.27–7.04)**
Family Structure				
Biological parents	132(82.50%)	145(90.06%)		
Single/step parent	28(17.5%)	16(9.94%)	0.062	1.86(0.97–3.56)
Family Conflicts				
No	121(76.10%)	146(91.25%)		
Yes	38(23.90%)	14(8.75%)	**0.001**	**3.09(1.57–6.10)**
Emotional Abuse				
No	110(68.75%)	146(94.19%)		
Yes	50(31.25%)	9(5.81%)	**<.0001**	**10.50(3.77–29.28)**

P-value and OR (95% CI) were obtained from the multivariate logistic regression model that simultaneously included family-environmental factors.

In Table 3, we observed a significant association between ADHD subjects and being physically inactive (OR = 1.68, 95% CI = 1.05–2.68, p = 0.03).

**Table 3:** Distribution of Lifestyle Factors of ADHD: Comparisons of ADHD Cases and Non-ADHD Control Subjects.

Characteristic	ADHD(n = 161)	Control(n = 161)	p-Value	OR (95% CI)
Domestic tobacco smoke				
No	94(58.75%)	98(61.64%)		
Yes	66(41.25%)	61(38.36%)	0.480	1.18(0.74–1.88)
Domestic alcohol consumption				
No	101(63.92%)	113(72.44%)		
Yes	57(36.08%)	43(27.56%)	0.159	1.40(0.88–2.24)
Physical activity				
>3 days	68(43.87%)	77(56.62%)		
≤3 days	87(56.13%)	59(43.38%)	**0.030**	**1.68(1.05–2.68)**
TV viewing				
≤2 hours per day	142(89.87%)	126(90.00%)		
>2 hours per day	16(10.13%)	14(10.00%)	1.000	1.00(0.48–2.10)
Internet usage				
≤2 hours per day	117(74.05%)	106(68.83%)		
>2 hours per day	41(25.95%)	48(31.17%)	0.634	1.20(0.56–2.53)
Accidental injury				
No	127(79.87%)	133(84.18%)		
Yes	32(20.13%)	25(15.82%)	0.338	1.30(0.73–2.33)
Daily dietary supplement intake				
No	117(74.05%)	106(68.83%)		
Yes	41(25.95%)	48(31.17%)	0.319	0.77(0.45–1.32)

P-value and OR (95% CI) were obtained from the multivariate logistic regression model that simultaneously included lifestyle factors.

The remaining variables in our analysis were not associated with ADHD status (Table 1, 2, 3).

### Stepwise Logistic Regression Models Predicting the Strongest Association between All Factors and ADHD

To eliminate multivariable interaction and multicollinearity, we performed a backward stepwise logistic regression based on the maximum partial likelihood estimates. In the final best-fit model, the associations between ADHD and maternal stress during pregnancy, maternal age at childbirth, paternal education, family conflicts, and physical activity which were once significant (Table 1, 2, 3) had changed to not significant and were removed from the final model.

In contrast, the ORs (Table 4) for the association between emotional abuse and ADHD (OR = 11.09, 95% CI = 2.15–57.29, P = 0.004), and the association between being a single child and ADHD (OR = 6.32, 95% CI = 2.09–19.14, P = 0.001), were largely unchanged from estimates obtained in the original model used in Table 2. Therefore these two variables were kept in the final best fit model.

**Table 4:** Associated Factors Identified in Backward Stepwise Logistic Regression Model.

Variables	Estimate^a^	Standard Error	Wald Test	P-Value	OR^b^ (95% CI)
Single child	1.84	0.57	10.64	0.001	6.32(2.09–19.14)
Emotional abuse	2.41	0.84	8.25	0.004	11.09(2.15–57.29)

Variables entered into the Model: sex, age, maternal age at childbirth, maternal stress during pregnancy, pregnancy induced hypertension, maternal education, paternal education, single child, family structure, family conflicts, emotional abuse, domestic tobacco smoke, domestic alcohol consumption, physical activity, TV viewing, internet usage, accidental injury, and dietary supplement intake.

a values are the estimated unstandardized regression coefficients.

b OR indicates likelihood of an ADHD.

## Discussion

In this study, we examined the effects of family-environmental factors in pair-matched ADHD cases and normal controls in a population of Chinese children, 5 to18 years of age. The results show that having suffered emotional abuse and being a single child were likely to have strong associations with ADHD. The ADHD subjects were 11 times more likely to suffer from emotional abuse as compared to the normal controls.

### Emotional Abuse

Recently, family-environmental factors such as abnormal intra-familial relationships, lack of emotional warmth towards the child, child maltreatment (physical/emotional), frequent arguments and fights between adults in the family, ambiguous communication patterns, parental separation/divorce and isolated family units, showed a trend towards an increasing risk for having a child with ADHD-related disorders, as reported previously in numerous studies. Poor parenting and conditions such as negative, inconsistent and detached parenting have been repeatedly reported as a risk factor for ADHD children [11], [38]. Pires et al. described that negative family relationships are associated with symptoms of ADHD, and children who suffered verbal abuse from their mother had prevalence 3.7 times higher than the ones not exposed to this situation in the last year [39]. Bandou et al. suggested that child abuse and maternal psychiatric disorders are significant risk factors in influencing the development of comorbid disruptive behavior disorders in offspring [12]. Also, Ouyang et al. indicated a significant association between inattentive ADHD symptoms and each type of child maltreatment [40]. Consistent with previous investigations, our present data confirms the findings of an association between ADHD and emotional abuse in Chinese children. We also found that the association is not influenced by other confounding factors.

There is a growing body of literature that supports the claim that psychological mistreatment is just as damaging as physical or sexual abuse, and child neglect [41]–[43]. Association between ADHD and child mistreatment has been reported for a variety of reasons [40]. The most important reason is that the behavior patterns found in children with ADHD disorders places those affected children at risk for parental mistreatment [40]. Extensive research on the parent-child interaction of children with ADHD reported a more stressful and conflicted family in those environments [10], [44]–[48]. Especially, child-rearing in Chinese societies is influenced by the Confucian ideology, which places emphasis on social norms and interpersonal harmony. Academic achievement is emphasized and dependence is encouraged. Due to the competition in joint entrance examinations for junior/senior high schools and universities being extremely strong, Chinese children are given more homework and spend more time receiving after-school tutoring [14], [16], [49]–[51]. As such, clinicians providing services to individuals with ADHD should be aware of the implications of co-occurring mistreatment and the associated risks.

### Single Child

Since the 1970s, China has had a one-child policy and family planning program. Today the total fertility rate is 1.6, which releases 24% more resources for the family and national investments [52]–[54]. China is becoming a small-family culture. A study showed that 73 to 75% of respondents in the wealthy Jiangsu province were satisfied with their one child regardless of sex, whereas in the poorer Anhui province, 58% were satisfied with an only boy, while only 31% were satisfied with an only girl [55]. Besides the sex ratio, old-age dependency may become a Chinese problem in the future due to the 4 (grandparents): 2 (parents): 1(child) phenomenon. In addition, another consequence of the one-child policy has been to create a spoiled “little prince” or “little princess” in some Chinese families. Information about the relationship between the single child status and the ADHD disorder in the Chinese population is lacking. This study revealed that having a single child in a family was associated with the risk of ADHD diagnosis. The association was not influenced by other confounding factors. The reason may be due to the single child receiving more parental concern, so they are more likely to be brought to medical professionals for assessment and diagnosis. Since we cannot explain clearly the mechanism that underlies the correlation between single child status and ADHD, further investigation for the causal relationship is needed.

### Other Family-environmental Factors

Previous studies have found that family-environmental factor such as family conflicts [56], [57] are associated with ADHD diagnosis. In contrast to these findings, the absence of statistically significant associations between the above factor and ADHD was unexpected in our study. The reason may be that the interactions between the related independent variables such as family conflicts and emotional abuse (correlation: r = 0.29, p<.0001), and family conflicts and family structure (correlation: r = 0.39, p<.0001) could influence family conflicts in the final best fit model. Previous works also found higher paternal education was associated with decreased risk for ADHD [24]. In our study, there is no relationship between paternal education and ADHD.

Previous reports have indicated that the biological factor of maternal stress during pregnancy is associated with ADHD [26], [27], [58]. In our study, there is no relationship between the above factor and ADHD. The reason might be recall bias, as the mothers who experienced life stress events during pregnancy may underreport it, which limits the statistical power in detecting a significant difference.

Previous studies also found that physical inactivity is associated with increased ADHD diagnosis in children [59]. We have not found the relationship between physical activity and ADHD in our study.

Further investigations are needed before definitive conclusions can be made about the possible effects of these potential factors on ADHD.

### Limitations

Several limitations should be considered in interpreting the results of this study. First, case-control studies offer only hints about causal models leading to the development of ADHD. The use of terms such as “prediction” and “risk” are not meant to imply causal or temporal relationships. Second, retrospective self-reporting of prenatal complications or family environment and lifestyle factors may be susceptible to recall bias. Observational measures of parent-child interactions may be necessary to understand moment-to-moment relationships between parent and child behavior patterns. Other variables such as in utero tobacco and alcohol exposure may be also susceptible to recall bias. Third, this study lacks of a comparison of the differences between ADHD subgroups. Future research may focus the relationship between family-environmental factors and ADHD subgroups, especially on the difference between subgroups. Fourth, all of our subjects were Chinese Han; the cohort was not a random sample of the Chinese population so potential selection biases cannot be fully ruled out. In addition, children living in foster families were not included, so the results of this study may not generalize to children with different socioeconomic or ethnic backgrounds. Future studies may be proposed to address these limitations.

### Conclusions

This hospital-based case-control study identified that emotional abuse and single child status are associated with ADHD children in China. The families and health care providers of children with ADHD should be aware of the implications of existing emotional abuse and the associated risks to these children. Appropriate parenting skills, such as proper supervision and prevention by increasing knowledge, need to be addressed for parents of children with ADHD, particular of single child families. Further investigations based on prospective, longitudinal birth cohorts are needed to explain the causal relationships between family-environmental factors and ADHD. Future studies may help to form the basis for successful intervention to prevent ADHD through reduction of family risk factors.
